# HEV-Capsid Protein Interacts With Cytochrome P4502C8 and Retinol-Binding Protein 4

**DOI:** 10.5812/kowsar.1735143X.768

**Published:** 2011-11-30

**Authors:** Quan Shen, Wen Zhang, Yanjun Kang, Yan Chen, Li Cui, Zhibiao Yang, Xiuguo Hua

**Affiliations:** 1 School of Agriculture and Biology, Shanghai JiaoTong University, Shanghai, China; 2 School of Medical Technology, Jiangsu University, Zhenjiang, Jiangsu, China; 3 Department of Zoonosis, Institute for Communicable Disease Control and Prevention, Chinese Center for Disease Control and Prevention, Beijing, China

**Keywords:** Hepatitis E Virus, Protein Interaction Mapping, Two-Hybrid System Techniques

## Abstract

**Background:**

Hepatitis E virus (HEV) is a major causative agent of acute clinical hepatitis in adults throughout much of Asia, the Middle East, and Africa. The lack of an efficient cell culture system for HEV has greatly limited our understanding of the mechanisms of infection, replication, and pathogenicity of this virus. The yeast two-hybridization system is considered to be an efficient method for determining protein-protein interactions and screening interactive proteins associated with host cells.

**Objectives:**

In order to identify the host-cell proteins interacting with the HEV-capsid proteins, a fragment of the HEV-capsid protein p239 (amino acids 368–606) was used as bait; human liver cDNA library was used as a source of host-cell proteins, and the screening was performed using the CytoTrap yeast two-hybrid system.

**Materials and Methods:**

The CytoTrap yeast two-hybrid system, which is also called Sos Recruitment System (SRS), was used to analyze the interaction of the p239 fragment with host-cell proteins.

**Results:**

We isolated 2 proteins, cytochrome P4502C8 (CYP4502C8) and retinol-binding protein 4 (RBP4) after 2 rounds of screening. Co-immunoprecipitation assays showed that both the proteins could bind in vitro to the HEV virion in HepG2 cells.

**Conclusions:**

CYP4502C8 and RBP4 screened from liver cDNA library using the CytoTrap yeast two-hybrid system interact with HEV capsid in vitro.

## 1. Background

Hepatitis E virus (HEV), the causative agent of hepatitis E, belongs to the family Hepeviridae. At least 4 major genotypes of HEV have been recognized; viruses with genotypes 1 and 2 are restricted to humans and associated with epidemics in developing countries, whereas those with genotypes 3 and 4 are zoonotic viruses and infect humans and several other animals in both developing and industrialized countries. A new HEV genotype was recently detected in a Norway rat in Germany [1]. The mechanisms of infection, replication, and pathogenesis of HEV remain unclear because of the unavailability of a suitable tissue culture system ; identifying the host-cell proteins that interact with this virus will help understand these viral mechanisms. The yeast two-hybridization system was considered to be an efficient method for determining protein-protein interactions and screening interacting proteins from host cells [2],[3]. Previous studies have shown that homodimers of the truncated HEV-capsid proteins E2 (amino acids 394–606) and p239 (amino acids 368–606) are the dominant antigenic determinants of HEV [4][5][6]. Furthermore, homodimers of p239 interact to form particles with a 23-nm diameter [5].

## 2. Objectives

Therefore, in the current study, we used a fragment of p239 as a bait to screen the proteins from the human liver cDNA library that interact with the HEV-capsid protein; the screening was carried out by using the CytoTrap yeast two-hybrid system.

## 3. Materials and Methods

### 3.1 Yeast Two-Hybrid System

The CytoTrap yeast two-hybrid system (Stratagene, CA), also known as the Sos Recruitment System (SRS), was used to screen the proteins from the host cells interacting with the HEV-capsid proteins [7]. This system generates fusion proteins whose interactions in the yeast cytoplasm activate the Ras-signaling pathway and induce cell growth. This system uses the temperature-sensitive mutant yeast strain cdc25H, which has a point mutation at amino acid residue 1328 of the CDC25 gene [8]. This mutation prevents the growth of the cdc25H strain at 37°C, but allows it to grow normally at room temperature. The CytoTrap system is based on the ability of the human Sos protein (hSos) to compensate for the defect in the CDC25 and to activate the Ras-signaling pathway in the yeast. Expression of hSos and its subsequent localization to the plasma membrane allows the cdc25H yeast strain to grow at 37°C. DNA encoding the protein of interest (bait protein) is cloned into the multiple cloning site of the pSos vector, following which a fusion protein is generated. Another protein of interest (i.e., the target protein) that is expressed as a fusion protein with a myristylation sequence is anchored to the plasma membrane. These fusion proteins are coexpressed in the cdc25H yeast strain, and the yeast cells are incubated at the restrictive temperature of 37°C. If the bait and target proteins interact with each other, hSos is recruited to the membrane, thereby activating the Ras-signaling pathway and allowing the cdc25H yeast strain to grow at 37°C.

### 3.2. Vectors

The fragment of HEV ORF2 coding for aa 394–606 was amplified by RT-PCR; the HEV ORF2 fragment was derived from the Chinese HEV strain SH-SW-zs1 (GenBank accession no. EF570133) with 5'-CAg gat CCC AGT TGT TTT ACT CAC GCC-3' (E2-sense) and 5'-CAg tcg ACC ACA GAA TGA GGT GCG AGG-3' (E2-antisense) (sites of the restriction enzymes BamHI and SalI are italicized). The amplified fragment was subcloned into the pSos vector. The recombinant Bait-E2 plasmid was sequenced to identify the correction. Other control vectors used in the yeast twohybrid screening were obtained along with the human liver cDNA library (Stratagene, USA).

### 3.3. Test of the Expression of Bait-E2 in Yeast Cells

Protein expression of the Bait-E2 plasmid in yeast cells was analyzed by western blot analysis. Briefly, 5 ml of the yeast cell culture transformed by the Bait-E2 plasmid was used for protein isolation after 3 days of incubation at room temperature. The yeast cells were pelleted by centrifuging the culture at 1000 g for 5 min, and then the pellet was resuspended into 200 µl of cell lysis buffer (140 mM NaCl, 2.7 mM KCl, 10 mM Na2HPO4, 1.8 mM KH- 2PO4, and 1% Triton X-100) containing freshly added protease inhibitors (1 mM phenymethylsulfonyl fluoride, 10 µg/ml aprotinin, 1 µM pepstatin A, 100 µM leupeptin, 1 µg/ ml chymostatin). The pellet resuspended in the buffer was vortexed with an equal volume of acid-washed glass beads (diameter, 0.5 mm, Stratagen) for 5 min at 4°C and then centrifuged at 12,000 g for 5 minutes. The lysate was collected and the supernatant was transferred into clean 1.5 ml screw-cap tubes. The analysis was performed by the standard western blot technique using mouse monoclonal anti-Sos antibody (Upstate, USA).

### 3.4. Screening

Before the screening, Bait-E2 was tested for absence of autoactivation by cotransforming it with pMyr Lamin C plasmid as previously described in the literature [9]. The screening was performed according to the manufacturer’s instructions of CytoTrap yeast two-hybrid system. Briefly, cdc25H yeast cells were cotransformed with the Bait-E2 plasmid and the cDNA library plasmid. The expression of the cDNA in the library plasmid was controlled by a galactose-inducible promoter. The transformants were grown on and selected from minimal-glucose plates and incubated for 4 to 6 days at 25°C. Subsequently, the colonies were replica-plated onto minimal-galactose plates and incubated at 37°C for 5 to 7 days. Positive colonies exhibiting efficient growth on the minimal-galactose plates at 37°C were isolated and tested for galactose-dependent growth at 37°C.

### 3.5. Isolation of pMyr cDNA Plasmid DNA From Yeast

Positive cDNA plasmids were isolated from yeasts by using E.Z.N.A.® Yeast Plasmid Kit (Omega Bio-Tek, USA) according to the manufacturer’s instructions. The plasmids were transformed into competent DH5a E. coli cells and then isolated for sequencing. Similarity searches for the sequences were carried out using Basic Local Alignment Search Tool (http://www.ncbi.nlm.nih.gov/BLAST/). In order to verify the specificity of the interaction between the bait and the target proteins, the bait and positive plasmids were re-transformed into yeast cells and then plated onto selective media. The transformants were tested for their ability to grow on SD/galactose agar plates at 37°C.

### 3.6. Immunoprecipitation Assays

Immunoprecipitation assays were performed to identify the interaction of the proteins in vitro. HepG2 cells, the permissive cell line for HEV, were maintained and cultured in Dulbecco’s Modified Eagle’s Medium (DMEM, Invitrogen, USA) containing 10% fetal bovine serum (FBS). These cells were plated on a 100-mm plate and incubated for about 2 days to obtain a good monolayer. They were then washed with cold phosphate-buffered saline (PBS) for 3 times, and subsequently, 1 mL of radioimmunoprecipitation assay buffer was added to the cells. The cell lysate was obtained by centrifugation at 15,000 g for 30 min at 4°C after incubating the cells on ice for 5 minutes with periodic mixing. Before immunoprecipitation, 20 µl of Protein A + G Agarose (Tiangen, China) and 1 µg of normal IgG (both obtained from the same species) along with the detection antibodies were added to 200 µL of the cell lysate to pre-clear the lysate. The lysate was centrifuged at 1000 g for 5 min at 4°C after incubating them for 3 h, and the supernatant was transferred to fresh tubes. We added 30 µL of Protein A+G Agarose and 0.4 µg of rabbit polyclonal antibody against recombinant human RBP4 (Sino Biological Inc., USA) or anti-HEV polyclonal antibody (Millipore, USA) to the lysate. The cell lysate mixture was then incubated overnight with gentle shaking at 4°C. The immunoprecipitates were collected by centrifuging the cell lysate mixture at 1000 g for 5 min at 4°C and then washed with 1.5 mL PBS 4 times; they were then resolved by sodium dodecyl sulpfate-polyacrilamide gel electrophoresis (SDS-PAGE) and detected by western blotting. Briefly, the samples were heated at 95°C for 5 minutes. After the gel was run, the proteins were transferred onto PVDF membranes (Millipore, Japan) by using Semi-dry Blotters (Hoefer Inc., USA) at 75 mA for 1 h. The membranes were blocked for 30 min by suspending them in 1X PBS containing 5% non-fat dry milk and 0.1% Tween-20 on a shaker at room temperature; they were further incubated overnight in HEV viral suspension at 4°C. After the membranes were washed 3 times with PBS, they were incubated with the secondary antibody (Millipore, Japan) at a dilution of 1:1000 for 2 h at room temperature. Subsequently, the bound antigen was detected by using the 3, 3', 5, 5'-Tetramethylbenzidine substrate (Tiangen Biotech, China).

## 4. Results

### 4.1. Bait-E2 Was Suitable for CytoTrap

**Figure 1:**
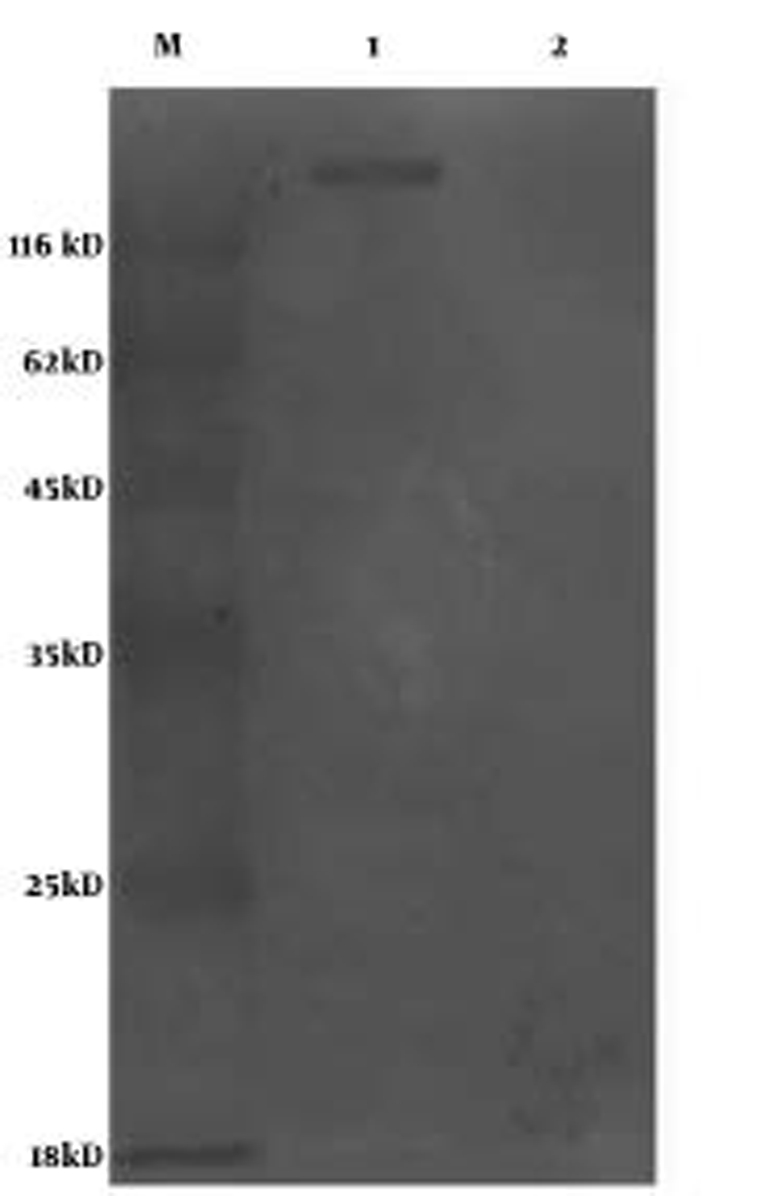
Analysis of the Expression of Bait-E2 in Yeast Cells by the Western Blot Method[Lane 1, yeast cells transformed with bait-e2; lane 2, yeast cells transformed with the pSos vector.]

After the sequencing, Bait-E2 was transformed into cdc25H yeast cells to detect the expression of the plasmid by performing western-blot analysis. The results showed that the ORF2 E2 fragment of HEV can fuse with the gene encoding hSos, following which this protein could be expressed(Figure 1), and that cdc25H yeast cells cotransformed with the positive plasmid pMyr Lamin C cannot grow in either SD (–UL)/Glucose or SD (–UL) Galactose media, which indicates that the bait plasmid does not show auto-activation.

### 4.2. Yeast Two-Hybrid Screening

**Figure 2:**
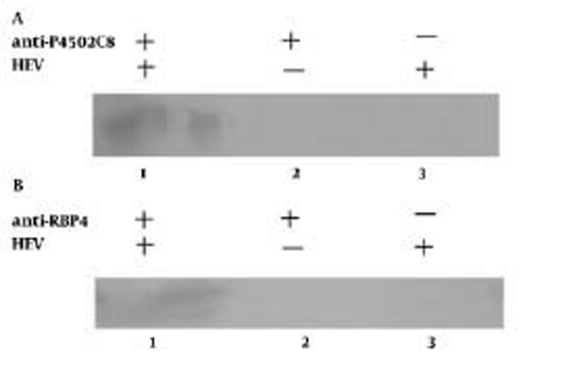
Immunoprecipitation and Western-Blot Assay for Detection of the Relationship Between HEV and Cytochrome P4502C8 (A) or RBP4 (B).

We identified 3 positive clones from the human liver cDNA library after 2 rounds of screening; 2 of these 3 clones had the same nucleotide sequence, and thus, we identified 2 distinct sequences. Similarity searches for these 2 sequences indicated that one shared 100% nucleic acid identity with the gene encoding human cytochrome P4502C8 (CYP4502C8) and the other shared 100% nucleic acid identity with the gene encoding Homo sapiens retinol binding protein 4 (RBP4). To eliminate the chance of a false-positive clone and to identify the reactions, each of those 2 plasmids was recotransformed into cdc25H yeast cells together with Bait-E2. We observed that both of the yeast cells grew in SD/glu (–UL) agar plate at 25°C and SD/gal (–UL) at 37°C, but did not grow in SD/ glu (–UL) at 37°C, which indicates that the 2 proteins interacted with the HEV-capsid fragment. The immunoprecipitation assays showed that both CYP4502C8 and RBP4 had immunoprecipitation reactions with HEV virion in HepG2 cells(Figure 2).

## 5. Discussion

Hepatitis E is a major public health disease in many developing countries of Asia and Africa, and it also occurs sporadically in some industrialized countries [10][11][12]. HEV is known to be a zoonotic virus, and some of its genotypes (genotype 3 and 4) are transmitted freely between swine and humans in some developing and developed countries [13][14][15]. However, the lack of an efficient cellculture system for HEV greatly hampers detailed analysis of the replication cycle of the virus in infected cells, because of which many important questions are difficult to resolve [16]. Understanding the interactions between the virus and proteins from host cells are efficient ways to analyze viral infection, replication, and other processes associated with infection by this virus. The yeast two-hybridization system is considered as an efficient method for screening interactive proteins involved in the pathogenesis of HEV. We isolated 2 proteins, CYP4502C8 and RBP4, after performing 2 rounds of screening. Both the proteins were further analyzed by the immunoprecipitation assay, and they were found to interact with the HEV virion. The CYP450 superfamily is a large and diverse group of enzymes. The function of most CYP enzymes is to catalyze the oxidation of organic substances. Research indicates that the HEV-capsid protein interacts with CYP2A6 and decreases its coumarin 7-hydroxylation activity [17]. Previous studies indicate that the expression of CYP2A6 markedly increases in hepatocytes immediately adjacent to areas of fibrosis and inflammation in sections of liver infected with HBV or hepatitis C virus (HCV), and that it is impaired in acute hepatitis A infection [18][19]. Recent investigations have shown that apart from viral factors, CYP2E1 polymorphism might be associated with the increased risk of liver diseases, including that by HEV in northeast India [20]. In the current study, HEV-capsid proteins interacted with CYP4502C8, an isoenzyme of CYP2A6 and CYP2E1. Taken together, the results suggest that various human cytochrome P450 isoforms probably are closely related to HEV. Therefore, we conclude that HEV may infect host cells by interacting with the cytochrome P450 family proteins and result in the activation of these enzymes. However, further extensive research is required to identify if and how the virus affects the enzyme activities. Retinol-binding protein 4 (RBP4) is the only specific transport protein for the circulation of retinol (vitamin A), and its main function is thought to be the delivery of retinol to tissues [21]. Studies have shown that serum RBP4 level is correlated with hepatocellular injury in patients with nonalcoholic fatty liver disease (NAFLD), and that it is also a new marker for virus-induced steatosis in patients infected with HCV having genotype 1 [22][23]. Recently, Kwon et al found that the level of serum RBP4 determines disease severity in patients with chronic liver disease [24]. However, thus far, the relationship between hepatitis E and RBP4 remains unknown, and the relationship between HEV and RBP4 and the effect of this interaction on disease severity is unclear.
